# Preliminary evaluation on the efficiency of the kit Platelia Dengue NS1 Ag-ELISA to detect dengue virus in dried *Aedes aegypti*: a potential tool to improve dengue surveillance

**DOI:** 10.1186/1756-3305-7-155

**Published:** 2014-04-01

**Authors:** Gabriel Sylvestre, Mariana Gandini, Josélio MG de Araújo, Claire F Kubelka, Ricardo Lourenço-de-Oliveira, Rafael Maciel-de-Freitas

**Affiliations:** 1 Laboratório de Transmissores de Hematozoários, Instituto Oswaldo Cruz, Fiocruz, Rio de Janeiro, RJ, Brazil; 2 Laboratório de Imunologia Viral, Instituto Oswaldo Cruz, Fiocruz, Rio de Janeiro, RJ, Brazil; 3 Laboratório de Biologia Molecular de Doenças Infecciosas e do Câncer, Departamento de Microbiologia e Parasitologia, Universidade Federal do Rio Grande do Norte, Natal, RN, Brasil

**Keywords:** *Aedes aegypti*, Dengue, Surveillance, NS1, ELISA, RNA, PCR

## Abstract

**Background:**

Surveillance is a critical component of any dengue prevention and control programme. Herein, we investigate the efficiency of the commercial kit Platelia Dengue NS1 Ag-ELISA to detect dengue virus (DENV) antigens in *Aedes aegypti* mosquitoes infected under laboratory conditions.

**Methods:**

Under insectary conditions, four to five day-old mosquitoes were orally challenged with DENV-2 titer of 3.6 x 10^5^ PFU equivalent/ml, incubated for 14 days and then killed. At ten time-points following mosquito death (0, 6, 12, 24, 72, 96, 120, 144 and 168 h), i.e., during a one-week period, dried mosquitoes were comparatively tested for the detection of the NS1 antigen with other methods of detection, such as qRT-PCR and virus isolation in C6/36 cells.

**Results:**

We first observed that the NS1 antigen was more effective in detecting DENV-2 in *Ae. aegypti* between 12 and 72 h after mosquito death when compared with qRT-PCR. A second round involved comparing the sensitivity of detection of the NS1 antigen and virus isolation in C6/36 cells. The NS1 antigen was also more effective than virus isolation, detecting DENV-2 at all time-points, i.e., up to 168 h after mosquito death. Meanwhile, virus isolation was successful up to 96 h after *Ae. aegypti* death, but the number of positive samples per time period presented a tendency to decline progressively over time. From the 43 samples positive by the virus isolation technique, 38 (88.4%) were also positive by the NS1 test.

**Conclusion:**

Taken together, these results are the first to indicate that the NS1 antigen might be an interesting complementary tool to improve dengue surveillance through DENV detection in dried *Ae. aegypti* females.

## Background

Dengue viruses belong to the Flaviviridae family as four antigenically related but distinct serotypes designated DENV-1, −2, −3 and −4. Dengue is an impressive, worldwide spread vector borne disease with higher incidence in the tropics due to the distribution of its vector, the *Aedes aegypti* mosquito. There are an estimated 50 million dengue infections annually, and remarkably, around 2.5 billion people live in dengue endemic countries [1,2].

Dengue epidemics have a seasonal trend and are often associated with the introduction of a new serotype in an immunologic naïve human population [3]. In Brazil, all serotypes have been co-circulating since 2009 when DENV-4 was registered in the North region and dispersed rapidly throughout the country [4,5]. Largely due to its size and climate, Brazil has the highest numbers of dengue cases in the Americas, accounting for more than 65% of cases in Latin America in the period between 2001–2010 [6].

Dengue serodiagnosis is usually based on anti-DENV IgM and IgG detection measured by MAC-ELISA and IgG-ELISA. However, specific antibody detection has a limitation concerning the acute phase of the disease, since the detection of DENV IgM takes 3 to 5 days and anti-DENV IgG requires 1 to 14 days, depending upon primary or secondary dengue disease [7].

Viral genome or viral proteins can be detected during the acute phase, such as NS1, a non-structural protein that may be involved in dengue replication [8,9]. NS1 can be found either as a membrane-associated protein in infected cells or as a secreted form, both reported to be immunogenic [8,9]. High NS1 levels were observed during the dengue acute phase in serum by antigen capture ELISAs, both in primary and secondary infections and up to the ninth day after onset of the symptoms [9]. Recently, the Brazilian Ministry of Health has established the NS1 capture commercial kits for the laboratorial diagnosis of dengue infections in sentinel clinics [10].

Surveillance is a critical component of any dengue prevention and control campaign, because it provides the necessary information for risk assessment and programme guidance, including epidemic response and programme evaluation. Integrated dengue surveillance should include the rapid detection of human infection supported by valid clinical and laboratory diagnosis, vector surveillance as well as monitoring of environmental and social risk factors for dengue epidemics to ensure that increased dengue transmission is detected early and that the response is rapid and appropriate. Viral surveillance to detect invasion of a new or re-introduction of a long time absent serotype is expensive and would be inefficient if laboratory confirmation of DENV were not rapid. Therefore, a delay in vector control activities caused by prolonged laboratory confirmation may contribute dramatically to increased dengue incidence and geographic viral spread [11].

One potential but underestimated approach would focus on monitoring dengue virus in field collected mosquitoes rather than in human sera. If dengue viruses are detected in mosquitoes earlier, rather than in symptomatic dengue patients, vector control activities may be intensified in those areas where dengue infected mosquitoes were collected. This early detection of dengue viruses may change the course of dengue epidemics, minimizing the morbidity and mortality during severe outbreaks.

On the other hand, the establishment of a large-scale mosquito collection routine for dengue virus surveillance imposes some logistical issues. Active surveillance of dengue infected mosquitoes in large geographical areas may be achieved with mosquito traps. But, according to the available molecular assays, collected mosquitoes would have to be processed either live or kept frozen until testing to avoid or reduce viral genome degradation [12,13]. For instance, it is commonly assumed that under unfavorable ambient conditions RNA is susceptible to rapid degradation by the activity of ribonucleases and microorganisms involved in cell decomposition rendering virus detection problematic after a relatively short period [14]. Therefore, the rationale of a dengue virus surveillance in field mosquito populations would only be justified if dengue detection were effectively performed in dead and dried female adult mosquitoes collected from appropriate traps.

Dengue control would benefit if a rapid and effective tool for virus surveillance were incorporated into the routine of dengue control programs. Our aim is to evaluate the efficiency of a NS1 antigen ELISA assay as a surveillance tool to detect DENV in dried mosquitoes. Accordingly, our objective was to compare the efficiency of the NS1 commercial kit Platelia Dengue NS1 Ag-ELISA developed by Bio-Rad against other diagnostic techniques, such as qRT-PCR, RT-PCR and virus isolation, in detecting dengue virus in previously DENV-2 infected dried mosquitoes.

## Methods

### Mosquitoes

*Ae. aegypti* Paea, a laboratory colony initiated with mosquitoes collected in French Polynesia in 1994, were dengue infected. Based on the observation of 15–20 generations per year in our laboratory, the Paea strain has been maintained for approximately 250–300 generations. This strain is highly susceptible to oral dengue infections and is routinely adopted as a control of vector competence experiments under laboratory controlled conditions [15,16]. Larvae were reared on yeast extract and raised in plastic basins at 25 ± 3°C. After emergence, adults were maintained in insectary conditions at 27 ± 2°C, 75 ± 5% relative humidity with a 12–12 h light–dark photoperiod in cages of 45 cm^3^ and allowed to mate. They were fed *ad libitum* with cotton soaked with 10% sucrose up to 24 h before the infective blood meal.

### Virus strain, stock preparation and titration

Dengue virus serotype 2 (DENV-2/strain Thailand/16681/1984) was provided by Dr. S. Halstead (Naval Medical Research Center, USA). The virus stock was obtained from four passages in Green monkey kidney (Vero) cell clone (ATCC CCL81). The cell culture was grown as monolayers and maintained at 37°C with 5% CO_2_ in 199 medium with Earle’s salts buffered with sodium bicarbonate and supplemented with 5% inactivated fetal calf serum (iFCS) and penicillin-streptomycin. Culture monolayers were infected with a 10-fold diluted viral inoculum without iFCS and incubated at 37°C for 90 minutes. After 7 days of infection, the supernatant was harvested and then centrifuged at 400 g for 10 minutes for cellular debris removal. The virus stock was stored at −70°C with 30% iFCS. An uninfected flask was also maintained and the supernatant was collected to be used as a mock inoculum. The virus was titrated by quantitative reverse transcriptase polymerase chain reaction (qRT-PCR) and by serial dilution cultures in microtiter plates and detected by indirect imunofluorescence (IFA) as previously described by Miagostovich *et al*. [17] and Schoepp and Beaty [18], respectively.

### Oral infection of mosquitoes with DENV-2

Four to five days after emergence, inseminated *Ae. aegypti* females were placed into small cylindrical plastic cages, with no access to sugar. About 36 h later, a DENV-2 infectious blood meal was offered which contained one ml of supernatant of infected cell culture added to 2 ml of washed rabbit erythrocytes. The infectious blood-meal was heated to 37°C and provided to the mosquitoes in an artificial membrane feeding apparatus [19]. Mosquitoes were allowed to feed for 25 min on infectious blood that contained a viral titre of 3.6 x 10^5^ PFU equivalent/ml. The same procedure and apparatus fed control mosquitoes, but these received a non-infectious blood meal, with 1 ml of uninfected cell culture supernatant. All fully engorged females were incubated at 27 ± 1°C, in small cardboard cages, 10–15 females per cage, with sucrose.

### Experimental design

Fourteen day post-infection (dpi) females were killed by vigorous shaking of the cardboard cages. Our intention was to simulate as much as possible a natural death rather than freezing or using chemicals, such as ethyl acetate, that may produce any additional damage to mosquito cells and dengue virus genome. Orally infected and control dead mosquitoes were then left to dry in Petri dishes and sampled to be tested according to the experiments described below.

Firstly, we compared the effectiveness of Platelia Dengue NS1 Ag-ELISA with qRT-PCR to detect DENV infection in a batch of 120 *Ae. aegypti* simultaneously challenged with DENV-2. Lots of 10 dried mosquitoes were sampled at 0, 6, 12, 24, 48 and 72 h after mosquito death and analyzed with Platelia Dengue NS1 Ag-ELISA (N = 60) and qRT-PCR (N = 60). Mosquitoes were randomly assessed by one of each detection method. Briefly, we analyzed ten mosquitoes per technique per time point, simulating an environment where mosquitoes remained in relatively harsh conditions for three days after their death.

The second step consisted of a new oral infection experiment with the same mosquito population, dengue virus and infection protocol to comparatively assess DENV infection in mosquitoes by virus isolation in C6/36 *Ae. albopictus* cell culture and Platelia Dengue NS1 Ag-ELISA test. At this point, we infected 162 *Ae. aegypti* females and separated 18 specimens to be tested for dengue at one of each of the following time points: 6, 12, 24, 48, 72, 96, 120, 144 and 168 h. Roughly, half (n = 80) of the infected insects had their head removed immediately after death, individually squashed on glass slides and examined by IFA with serotype-specific monoclonal antibodies [20] to determine if the mosquitoes were infected at the time they were killed. Thus, at each time point we analyzed nine headless and nine full bodied mosquitoes. Because no difference was observed between these groups, we ignored this potential source of error. We increased the time points, simulating a scenario where mosquitoes remained at a natural environment for up to one week after death. This period is similar to those observed in several field experiments where mosquitoes are collected with adult traps on a weekly basis [21-23]. At each time point, each mosquito was ground in 200 μl of Leibovitz medium (L-15) and 50 μl of fetal calf serum. Each mosquito homogenate was divided into two aliquots for further comparison of effectiveness among tests: 50 μl were inoculated in C6/36 cell culture for virus isolation, and 50 μl was analyzed with the Platelia Dengue NS1 Ag-ELISA test.

### Cell culture and virus isolation

A C6/36 cell clone of *Aedes albopictus* (ATCC CRL-1660) was grown and maintained as monolayers at 28°C on Dulbecco’s modified medium (DMEM) buffered with sodium bicarbonate and supplemented with glutamine, penicillin-streptomycin and 5% iFCS. Virus isolation was performed by inoculation into the C6/36 *Aedes albopictus* cell line [24]. After 7 days of infection, the isolates were identified by IFA with serotype-specific monoclonal antibodies [17,20].

### RT-PCR assays

RT-PCR for DENV detection was carried out as described previously [25]. Briefly, consensus primers were used to anneal to any of the four DENV types and amplify a 511-bp product in a reverse transcriptase-polymerase reaction. A cDNA copy of a portion of the viral genome was produced in a reverse transcriptase reaction. After a second round of amplification (nested PCR) with type-specific primers, DNA products of unique sizes for each dengue virus serotype were generated.

### Platelia Dengue NS1 Ag-ELISA and cut-off threshold

The test is based on a one-step sandwich format microplate enzyme immunoassay to detect the DENV NS1 antigen in human serum or plasma. Detection of NS1 in mosquito samples was conducted in compliance with the manufacturer’s instructions (Bio-Rad). Briefly, the assay incorporates murine monoclonal antibodies for capture and revelation. The presence of NS1 in samples allows the formation of an immune-complex MAb-NS1-MAb/peroxidase. In a NS1 monoclonal antibody pre-coated plate, samples or controls were added (50 μl) to the same volume of sample diluent. After the addition of 100 μl of diluted conjugate, a 90 min incubation at 37°C ensued. After a six-time washing step, 160 μl of substrate was added to each well and incubated for 30 min at room temperature in the dark. A color development was characteristic of the immune-complex presence. The plate was read at 450 nm after reaction was stopped with 100 μl 1NH_2_SO_4_. Since we used this kit to detect DENV in mosquitoes, not in human sera as the original purpose of this kit, we added 2–3 known uninfected *Ae. aegypti* female as an additional negative control in each plaque assay. Thus, classification whether a sample was positive was based on the comparison of the OD of the sample to the OD of the cut-off control mosquitoes. The cut-off OD for the Platelia Dengue NS1 Ag-ELISA based on DENV-uninfected mosquitoes was 0.118.

### Quantitative real-time PCR

The concentration of viral RNA in each insect was estimated with a one-step RT-PCR with the ABI Prism® 7000 Sequence Detection System (SDS; Applied Biosys-tems, Foster City, CA, USA). Viral RNA from each mosquito was extracted with the QIAmp Viral RNA Kit (Qiagen Sciences, Maryland, MA, USA). The reaction mixture was prepared with the Taqman® One- Step RT-PCR Master Mix Kit (Applied Biosystems, Foster City, CA, USA). Samples were assayed in a 30 μl reaction mixture containing 8.5 μl of extracted RNA, 0.63 μl of 40 × Multiscribe enzyme plus RNAse inhibitor, 12.5 μl TaqMan 2 × Universal PCR Master Mix and 300 nM of each specific primer and fluorogenic probe. Primer sequences (DV2.U: 5′- AAG GTG AGA TGA AGC TGT AGT CTC-3′; DV.L1: 5′-CAT TCC ATT TTC TGG CGT TCT-3′) and probe (DV.P1: 5′- CTG TCT CCT CAG CAT CAT TCC AGG CA-3′) were obtained from Houng *et al*. [26] and designed for the 3′ noncoding sequences (3′NC). The TaqMan probe was labeled at the 5′ end with 5-carboxyfluorescein (FAM) reporter dye and at the 3′ end with 6-carboxy-N,N,N’,N’- tetramethylrho-damine (TAMRA) quencher fluorophore. The 5′ nucleaseTaqMan assay relies on the 5′ exonuclease activity of the Taq polymerase to free the reporter dye in the quenched probe. DENV-2 viral stocks were used as positive controls, while water and non-infected individuals were used as negative controls and included in every assay. The threshold cycle (Ct) represents the PCR cycle at which the SDS software first detects a noticeable increase in reporter fluorescence above a baseline signal. The quantitative Real-Time PCR assays were standardized with a 10-fold-dilution series containing 10^7^ RNA copies/ml. The number of viral RNA copies detected per sample was calculated with a standard curve from 10-fold dilutions of DENV-2 RNA, isolated from a known amount of local virus propagated in *Ae. albopictus* C6/36 cells, the titer of which was determined by plaque assay. Quantitative interpretation of the results was carried out by interpolation from the standard curve included in each independent run.

## Results

### Platelia Dengue NS1 Ag-ELISA and qRT-PCR

The Platelia kit and qRT-PCR methods showed equivalent efficiency to detect DENV up to 6 hours after death, with 60 to 70% positive individuals. For this strategy, distinct lots of 60 *Ae. aegypti* females previously fed with a blood meal containing DENV-2 were examined respectively by NS1 Ag-ELISA and qRT-PCR. Considering all sampling times, we detected DENV infection in 61 (50.8%) mosquitoes: 38 (63.3%) by the Platelia Dengue NS1 Ag-ELISA and 23 (38.3%) by the qRT-PCR (Table 1). Remarkably, NS1 detection displayed an overall superior sensitivity than qRT-PCR between 12 to 72 hs after mosquito death (Fisher exact test, P <0.0001). Accordingly, 25 dried females examined 12 to 72 hours after death were positive for DENV infection by NS1 ELISA, while only 10 were positive by qRT-PCR (Table 1). The qRT-PCR was highly efficient to detect dengue virus RNA in mosquitoes up to six hours after death, as 70% were positive by this method. At later time points, the positivity rates by qRT-PCR dropped to 40% at 12 and 24 h after mosquito death, to only 10% at 48 and 72hs after mosquito death (Table 1).

**Table 1:** ***Ae. aegypti *** **females positive for dengue infection; ranges of optical density and cycle threshold plus RNA copies using Platelia Dengue NS1 Ag-ELISA and qRT-PCR, respectively, in detecting DENV-2 between 0–72 hours after mosquitoes were killed and maintained at insectary temperatures**

**Time points (h)**	**DENV-2 detection (NS1)**		**DENV-2 detection (qRT-PCR)**		
	**Positives (%)**	**Optical density range**	**Positives (%)**	**Cycle threshold range**	**RNA copy range**
0	6 (60)	0.101 – 2.874	6 (60)	38.05 – 42.98	2278.06 – 14112.42
6	7 (70)	0.116 – 3.58	7 (70)	36.78 – 43.87	1641.77 – 22576.03
12	8 (80)	0.101 – 3.003	4 (40)	37.99 – 44.36	1367.33 – 14413.18
24	7 (70)	0.111 – 3.92	4 (40)	39.12 – 43.64	1946.2 – 9519.86
48	5 (50)	0.099 – 2.876	1 (10)	42.83	2410.76
72	5 (50)	0.156 – 3.071	1 (10)	40.36	6525.18
Total	38 (63.3)	–	23 (38.3)	–	–

**Figure 1:**
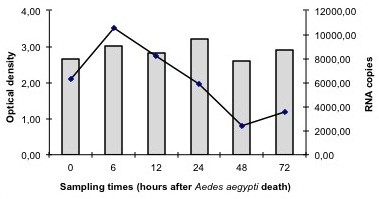
**Comparison between the median optical density revealed by the Platelia Dengue NS1 Ag-ELISA (bars) and the median number of RNA copies determined by qRT-PCR (line) of *Aedes aegypti *** **mosquitoes killed naturally and analyzed for dengue virus detection between 0–72 h after death.**

Intriguingly, optical density values of positive samples measured by NS1 ELISA exhibited a tendency to remain constant, while the number of RNA copies in qRT-PCR decreased over time (Figure 1). Furthermore, the cycle threshold of positive samples, i.e. the amplification cycle when dengue virus RNA was first detected above a baseline signal, presented a tendency of increase over time, which was expected.

### Platelia Dengue NS1 Ag-ELISA versus virus isolation

From the 80 *Ae. aegypti* females that had their heads removed immediately after mosquito death and examined by IFA, 78 (97.5%) were positive for DENV. We compared the success of NS1 Ag-ELISA and virus isolation in C6/36 cells in DENV infection detection in the same individual mosquito homogenate. We observed higher sensitivity with the Platelia Dengue NS1 Ag-ELISA rather than virus isolation in C6/36 cells (McNemar’s Chi-squared test = 31.75, P < 0.0001) (Table 2). The NS1 approach was able to determine DENV in 63 (39.4%) samples, while the cell culture inoculation of mosquito homogenates was positive in 32 (20%) samples (Table 2). The NS1 approach was successful in DENV detection at all time-points, i.e. between 6–168 h after mosquito death, even one week after mosquitoes were dead. Virus isolation was successful up to 96 h after *Ae. aegypti* death, but the number of positive samples per time period presented a tendency to decline progressively over time. From the 43 samples positive by the virus isolation technique, 38 (88.4%) were also positive by the NS1 test.

**Table 2:** **Percentage of positive samples per time points as well as the total for the two dengue detection methods in *Aedes aegypti *** **mosquitoes: virus isolation and Platelia Dengue NS1 Ag-ELISA**

**Time points (h)**	**Virus isolation**	**Platelia Dengue NS1 Ag-ELISA**
6	33.3 (6/18)	55.6 (10/18)
12	44.4 (8/18)	61.1 (11/18)
24	38.9 (7/17)	44.4 (8/17)
48	29.4 (5/17)	52.9 (9/17)
72	27.8 (5/18)	33.3 (6/18)
96	5.6 (1/18)	44.4 (8/18)
120	0.0 (0/18)	33.3 (6/18)
144	0.0 (0/18)	22.2 (4/18)
168	0.0 (0/18)	5.6 (1/18)
Total	20.0 (32/160)	39.4 (63/160)

## Discussion

Detection of DENV in dried *Aedes aegypti* mosquitoes collected from the field would provide a significant milestone for dengue surveillance. Herein, we evaluated the effectiveness of the NS1 Ag-ELISA in detecting DENV-2 in dried mosquitoes up to 21 days post-infection and one week after their death with subsequent maintenance under insectary conditions (27 ± 2°C, 75 ± 5% r. h.). This scenario simulated the harsh conditions of temperature and humidity frequently present in the field of tropical dengue endemic and epidemic areas. Since the NS1 approach overall presented higher efficacy than qRT-PCR, RT-PCR and virus isolation, especially after a prolonged mosquito death, it may be considered as a potential complementary tool to improve DENV surveillance, not only for detecting DENV in human serum but especially by detecting DENV infection in the mosquito population. If successful, the early detection of DENV in the mosquito population would afford greater efficiency of vector control activities in those areas with higher dengue prevalence. Therefore, the introduction of this surveillance practice in routine vector control programs, especially if used in combination with a diagnostic method that discriminates between DENV serotypes, would potentially reduce the burden of dengue outbreaks. An important point regarding the potential use of the NS1 antigen as tool to improve DENV surveillance would be to test its sensitivity to different DENV serotypes, especially in dengue endemic areas with co-circulation of the four serotypes.

One way to overcome such a challenge is to look for the presence of virus in dry mosquitoes. Likewise, live specimen collection would not be mandatory for DENV detection in mosquitoes. Arboviruses detection in dried mosquitoes has been successful in other systems, such as Japanese encephalitis virus (JEV)/*Culex tritaeniorhynchus*[27], JEV/*Culex vishinui* but also in DENV/*Ae. aegypti*[14,28,29]. These reports, however, employed different experimental designs that hampered potential comparisons between their results and ours. We orally challenged *Ae. aegypti* mosquitoes and killed them without freezing or using any substance that might influence DENV detection afterwards. Intrathoracic inoculations to infect *Ae. aegypti* females may have influenced virus dynamics and replication in the mosquito body, increasing the success of DENV detection [14,28,30]. Additionally, killing mosquitoes by freezing helped to preserve the virus genome, which also benefited virus detection [14,28,29]. Therefore, by orally infecting mosquito females without freezing, we believe our data may better represent some of the difficulties of DENV surveillance in *Ae. aegypti* natural populations.

Some commercial kits for the NS1 detection approach have been reported to detect DENV in *Ae. aegypti* mosquitoes. For instance, Tan *et al*. [31] demonstrated that the Dengue NS1 Ag Strip was efficient in detecting all DENV serotypes and was also able to detect DENV-2 up to 10 dpi when mosquitoes were stored at −80°C. DENV detection in *Ae. aegypti* mosquitoes was more efficient with the NS1 antigen than RT-PCR and virus isolation, but mosquitoes were infected by intrathoracic injection [30]. A dengue diagnostic rapid strip was able to detect the NS1 antigen in pools of 50 uninfected *Ae. aegypti* with a single infected specimen [32]. Conventional procedures for virus infection in vector mosquitoes are cumbersome for the routine monitoring of the infection status in mosquitoes and commercial kits seem to offer a rapid and sensitive alternative for detection of dengue in wild-caught mosquitoes.

In recent years, RNA copy number measured by quantitative real time-polymerase chain reaction has been used to measure dengue virus concentration *in vitro* and *in vivo*[33]. A recent study developed by Choy *et al*. (2013) showed that, although there was reasonably good correlation between the qRT-PCR and plaque assay, RNA copy number was 2 to 5 logs greater than infectious virus titers. These differences varied significantly depending on virus strain, viral platform, infectious virus assay, and viral growth phase [33].

The application of the NS1 antigen technique to detect DENV in wild mosquitoes may be a handy tool especially in cities where health systems do not have trained personal to perform more elaborate techniques such as RT-PCR assays, for instance. Even though, other methods to preserve biological material such as dead insect bodies from fungi and bacterial contamination, increased humidity, and degradation by proteases might be achieved with different storing methods such as silica gel during sample storage and transportation [34]. Taken together, our results reinforce the potential use of the DENV NS1 antigen by sensitive ELISA such as the commercial kit Platelia in detecting DENV in laboratory-reared infected mosquitoes as well as in field-collected specimens. The adoption of this approach in routine vector control programs would not only be valuable to improve dengue surveillance, but would most likely result in the reduction of outbreaks in endemic countries as well.

## Conclusion

The NS1 antigen was able to detect DENV in dried *Ae. aegypti* mosquitoes, with higher sensitivity than more traditional techniques such as qPCR and virus isolation in C6/36. These preliminary results are encouraging and suggest the NS1 approach might become an interesting complementary tool to improve dengue surveillance through DENV detection in dried *Ae. aegypti* females.

## Competing interests

The authors declare that they have no competing interests.

## Authors’ contributions

CK, RLO, RMF designed the study and acquired the funding; GS, MG and JMGA conducted the experiments, CK and RMF analyzed the data, GS, MG, JMGA and RMF wrote the manuscript. All authors read and approved the final version of the manuscript.
